# Change-of-Direction Speed in Firefighter Trainees: Fitness Relationships and Implications for Occupational Performance

**DOI:** 10.5114/jhk/161545

**Published:** 2023-04-20

**Authors:** Robert G. Lockie, Robin M. Orr, Fernando Montes, J. Jay Dawes

**Affiliations:** 1 Department of Kinesiology, California State University, Fullerton, Fullerton, CA, USA.; 2 Tactical Research Unit, Bond University, Robina, Qld, Australia.; 3 Los Angeles County Fire Department, Los Angeles, CA, USA.; 4 Tactical Fitness and Nutrition Lab, Oklahoma State University, Stillwater, OK, USA.

**Keywords:** agility, first responders, Illinois agility test, public safety, tactical

## Abstract

Change-of-direction (COD) speed and ability could assist a firefighter moving about the fire ground more efficiently. There has been limited investigations of COD speed in firefighter trainees, and what measures of fitness could contribute to faster performance in a test such as the Illinois agility test (IAT), which measures longer COD speed. This study analyzed archival data from 292 trainees (262 males, 30 females). The trainees completed the following fitness tests at their training academy: IAT, push-ups, pull-ups, leg tucks, 20-m multistage fitness test to measure estimated maximal aerobic capacity (VO_2max_), backwards overhead 4.54-kg medicine ball throw (BOMBT), 10-repetition maximum (10RM) deadlift, and a 91.44-m farmer’s carry with 2 x 18-kg kettlebells. Independent samples t-tests compared male and female trainees to determine the need to control for trainee sex in the analyses. Partial correlations, controlling for trainee sex, analyzed relationships between the IAT and fitness tests. Stepwise regression analyses controlling for trainee sex determined if any fitness test predicted the IAT. On average, male trainees outperformed females in all fitness tests (p ≤ 0.002). The IAT significantly related to all fitness tests (r = ±0.138–0.439, p ≤ 0.019), and was predicted by trainee sex, estimated VO_2max_, the 10RM deadlift, BOMBT, and the farmer’s carry (R = 0.631; R^2^ = 0.398; adjusted R^2^ = 0.388). The results indicate the trainees who are generally fit may perform well in a range of different fitness tests, including the IAT. Nonetheless, improving muscular strength (measured by the 10RM deadlift), total-body power (BOMBT), and metabolic capacity (estimated VO_2max_, farmer’s carry) could enhance COD speed in firefighter trainees.

## Introduction

Fighting fires can place unique physical demands on the individual firefighter when they are onsite with their team. Some examples of occupational tasks that may need to be completed by firefighters include carrying heavy equipment, operating hose lines, forcible entries, climbing stairs, raising ladders, crawling, searching, and rescuing casualties (Rhea et al., 2004; Sheaff et al., 2010). Firefighters must also complete these occupational tasks under load (which can include more than 20 kg of personal protective equipment and self-contained breathing apparatus) and in extreme (e.g., hot, wet, and smoky) environments, which makes the execution of these tasks more difficult (Lesniak et al., 2020; Walker et al., 2019). Based on the physical demands of firefighting, some studies have shown links between different aspects of fitness and the execution of job tasks in firefighters (Rhea et al., 2004). For example, while aerobic fitness underpins the majority of firefighting job tasks (Williams-Bell et al., 2009), especially when they need to be performed over an extended duration, muscular strength is required when performing tasks such as lifting and extending ladders (Stevenson et al., 2017). Consequently, attaining and maintaining a certain level of fitness is a priority for many fire agencies.

It is generally recommended that firefighter candidates should have an appropriate level of fitness before they officially commence their training to become a firefighter. In the USA, what aids in this process is the completion of a physical ability or agility tests prior to being accepted to a training academy. One example is the Candidate Physical Ability Test (CPAT), which is a nationally recognized test that replicates different firefighting job tasks (i.e., stair climb, hose drag, equipment carry, ladder raise and extension, forcible entry, search, rescue drag, and ceiling breach and pull) and quantifies a candidate’s ability to perform these tasks (Firefighter Candidate Testing Center, 2021). The design and time restrictions (the CPAT must be completed in 10 min and 20 s) of the CPAT challenges numerous aspects of fitness that a candidate must develop on their path to becoming a firefighter, including anaerobic and aerobic capacities (Sheaff et al., 2010; Williams-Bell et al., 2009).

An aspect of anaerobic capacity that has yet to be analyzed in detail in firefighter trainees is change-of-direction (COD) speed. COD speed is the foundation and the physical component of agility (Lockie et al., 2019), which is the ability to change direction in response to a stimulus (Sheppard and Young, 2006). Linear speed, movement technique, muscular strength and power, and deceleration and acceleration capabilities comprise COD speed (Ari et al., 2021; Fernandez-Fernandez et al., 2022; Lockie et al., 2016, 2019; Sheppard and Young, 2006). In occupational testing, COD speed tests are often used to measure multiple qualities (i.e., linear and COD speed in combination with lower-body power) (Cesario et al., 2018; Lockie et al., 2018). COD actions can be incorporated in certain physical ability tests for firefighters. As an example, the Biddle Physical Ability Test (BPAT), which is performed by firefighter candidates in California in the U.S., includes sections that require candidates to weave between markers while moving as fast as possible (Santa Ana College Fire Technology Department, 2021). COD ability could also assist a firefighter navigating quickly through unpredictable conditions for both urban and wildland firefighters. Running speed, strength, power, and dynamic stability (Fernandez-Fernandez et al., 2022; Lockie et al., 2016, 2019) are all needed for COD speed, and these fitness qualities should also benefit a firefighter moving through and keeping safe in austere conditions. It should be noted that the COD tasks required of firefighters typically have novel aspects when compared to those performed in sports (e.g., load carriage, unpredictable terrain, manipulation of equipment such as hoses and ladder) (Santa Ana College Fire Technology Department, 2021). Nonetheless, measurement of COD speed in firefighter trainees could provide a strength and conditioning coach with knowledge of the underlying capacity of the trainee to perform tasks that require direction changes. This information can then be used to develop training programs, especially for trainees lacking in this quality.

There has been some analysis of COD speed in firefighter trainees. For example, the Illinois agility test (IAT) has been used to assess COD speed in public safety occupations (Adams et al., 2014; Lockie et al., 2022). Specific to firefighter trainees, Lockie et al. (2022) provided normative data for the IAT performed by 237 males and 31 females from one fire department. The time for the IAT in the sample from Lockie et al. (2022) ranged from 16.0 s to 32.0 s. This time is indicative of a longer COD speed test (i.e., duration greater than 6 s) (Lockie, 2019; Lockie et al., 2019), which may place greater stress on an individual’s metabolic capacity (Vescovi and McGuigan, 2008). Furthermore, in college-aged men and women, Lockie et al. (2019) found that a faster 20-m sprint, better vertical, standing broad, and lateral jumps, and greater relative strength measured by the isometric midthigh pull all related to a faster IAT (correlation coefficient [*r*] = ±0.45–0.71). This would suggest that similar to previous research (Cesario et al., 2018; Lockie et al., 2018), the IAT as a COD speed measure could provide some indication of multiple fitness qualities in firefighter trainees. It is important to investigate COD speed specifically in firefighter trainees as they likely have different skill and physical fitness capabilities than college-aged civilians. This is especially true given the need for trainees complete firefighter-specific tests such as the CPAT prior to being accepted to a fire training academy (Sheaff et al., 2010; Williams-Bell et al., 2009). However, there has been no research detailing what fitness characteristics could contribute to faster COD speed in this population.

Therefore, the purpose of this study was to determine the relationships between different measures of fitness with the IAT in firefighter trainees. A further aim was to determine if any of the fitness variables predicted IAT performance. This study used pre-existing data recorded by fire department training staff, and the fitness testing battery featured a range of different assessments (Lockie et al., 2022). It was hypothesized that measures of muscular strength, power, and anaerobic capacity would correlate and predict the IAT in firefighter trainees. The results from this study would not only have application for firefighter trainees and those involved in physically demanding occupations, but also any athlete that requires COD speed in their sport.

## Methods

### Participants

Deidentified archival data from six academy classes from one fire department in the USA was released with consent to the researchers. The sample included data from 292 trainees (262 males, 30 females). All trainees were over 18 years of age, and had completed a pre-placement medical evaluation (Los Angeles County Fire Department, 2018). Specific age, height, and body mass data were not provided to the researchers, which has happened in other first responder studies (Butler et al., 2013; Lockie et al., 2022). One reason these variables may not be collected in these populations is to avoid any allegations of preferential hiring and retention based on age, body size and sex, based on the assumption that they are not job-related (Cooper Institute, 2014; Roehling, 1999). Inclusion criterion for a trainee in this study was available data for all the fitness tests. The exclusion criteria were clearly incorrect data through entry error or missing data points for any test. Based on the archival nature of this analysis, the California State University, Fullerton ethics committee approved the use of pre-existing data (HSR-17-18-401). The research conformed to the recommendations of the Declaration of Helsinki.

### Procedures

The procedures for data collection have been described by Lockie et al. (2022), and the reader is directed to this manuscript to review the specifics of the design and administration for each test. To briefly explain, all fitness tests were completed by trainees as part of their employment requirements within one 90-min physical training session for all classes. The testing sessions for all six academy classes were overseen by the same fire department staff member and occurred outdoors at the department’s training facility in the early morning (~6:00 am). Eight tests were performed by the trainees, and the testing battery was designed to measure a variety of fitness qualities (Lockie et al., 2022). Firstly, the IAT was used to measure COD speed (Lockie et al., 2019). Maximal push-ups performed in time with a metronome at a cadence of 80 beats per minute was utilized to determine upper-body muscular endurance (Lockie et al., 2022). Maximal pull-ups provided a metric for upper-body pulling strength (Lockie et al., 2018, 2022). Leg tucks were included to measure grip, arm, shoulder, and trunk muscle strength (Lockie et al., 2022). The 20-m multistage fitness test was performed by trainees and the number of completed shuttles was used to estimate maximal aerobic capacity (VO_2max_) in milliliters per kg of body mass per minute (ml•kg^–^^1^•min^–^^1^) (Ramsbottom et al., 1988). The backwards overhead medicine ball throw (BOMBT) with a 4.54-kg (10 lbs) medicine ball was performed to assess combined upper- and lower-body power (Stockbrugger and Haennel, 2003). A 10-repetition maximum (10RM) deadlift was completed to provide a strength metric (Lockie et al., 2022). Lastly, a farmer’s carry was included in the testing battery to gauge a trainee’s ability to perform loaded carries (Lockie et al., 2022). The farmer’s carry involved the trainee carrying 2 x 18-kg kettlebells in each hand four times up-and-back over a distance of 22.86 m (25 yards).

The tests were completed in the order stated, and as detailed by Lockie et al. (2022). Testing was not completed in the order relative to National Strength and Conditioning Association recommendations (McGuigan, 2015). However, this was standard practice within the department and all data measured were used for record. Fire academies have challenges relative to staff, space, time, and equipment (Lockie et al., 2022), such that the testing protocol was adapted relative to this situation.

### Statistical Analysis

All statistical analyses were computed using the Statistics Package for Social Sciences (version 28.0; IBM Corporation, NY, USA), and the data analysis was adapted from previous research (Lockie et al., 2019). Descriptive statistics (mean ± standard deviation [SD]) were calculated for each variable. Independent samples *t*-tests (*p* < 0.05) were used to ascertain any between-sex differences in the fitness tests to confirm the use of trainee sex as a control variable. This was done as previous first responder research has shown differences between males and females in fitness assessments (Cesario et al., 2018; Lockie et al., 2022). Effect sizes (*d*) were also calculated for the between-sex comparisons; the difference between the means was divided by the pooled SD (Cohen, 1988). The strength of *d* was defined as: <0.2 = trivial effect; 0.2–0.6 = small effect; 0.6–1.2 = moderate effect; 1.2–2.0 = large effect; 2.0–4.0 = very large effect; and ≥4.0 = extremely large effect (Hopkins, 2004). Following the confirmation of sex differences, partial correlations (*r*) controlling for trainee sex (*p* < 0.05) were used to calculate relationships between the IAT and the other fitness tests. Correlation strength for relationship prediction was delimited as: an *r* between ±0–0.3 = small; ±0.31–0.49 = moderate; ±0.5–0.69 = large; ±0.7–0.89 = very large; and ±0.9–1 = near perfect (Hopkins, 2006). A stepwise regression analysis (*p* < 0.05), with trainee sex as a control variable, was then conducted to determine if any fitness test predicted the IAT.

## Results

The descriptive data for all, male, and female trainees are shown in Table 1. Male trainees significantly outperformed female trainees in all tests, with the effect size strength ranging from small (push-ups) to very large (BOMBT). The between-sex difference for the IAT had a large effect. These results confirmed the need to control for trainee sex in the correlation and stepwise regression analyses. There were significant relationships between the IAT and all the other fitness tests (Table 2). All relationships indicated better performance in the fitness tests related to a faster IAT, with the correlation strengths ranging from small-to-moderate. The stepwise regression data are displayed in Table 3, including the *R, R^2^*, adjusted *R^2^, F* value, *p* value, and beta coefficients for each variable in the predictive relationships. All predictive relationships were significant at *p* < 0.01. The IAT was predicted by trainee sex, estimated VO_2max_, the 10RM deadlift, BOMBT, and the farmer’s carry, with an explained variance of 39%.

**Table 1:** Descriptive data (mean ± SD) and between-sex comparisons for all, male, and female firefighter trainees from one department for the Illinois agility test (IAT), metronome push-ups, pull-ups, 4.54-kg backwards overhead medicine ball toss (BOMBT), leg tuck, estimated maximal aerobic capacity from the 20-m multistage fitness test (estimated VO_2max_), 10-repetition maximum (10RM) deadlift, and the farmer’s carry.

Variable	All(N = 292)	Males(n = 262)	Females(n = 30)	*p*	*d*
IAT (s)	18.40 ± 1.19	18.26 ± 1.12	19.63 ± 1.00	<0.001	1.29
Push-ups (no.)	62.21 ± 22.75	63.56 ± 22.29	50.33 ± 23.68	0.002	0.58
Pull-ups (no.)	11.66 ± 6.43	12.42 ± 6.17	5.03 ± 4.75	<0.001	1.34
BOMBT (m)	9.56 ± 1.69	9.90 ± 1.38	6.58 ± 1.15	<0.001	2.61
Leg Tuck (no.)	11.99 ± 5.88	12.69 ± 5.56	5.87 ± 5.09	<0.001	1.28
Estimated VO_2max_ (ml•kg^–1^•min^–1^)	46.09 ± 5.93	46.60 ± 5.83	41.66 ± 4.94	<0.001	0.91
10RM Deadlift (kg)	143.31 ± 15.24	145.55 ± 12.81	123.73 ± 20.24	<0.001	1.29
Farmer’s Carry (s)	28.86 ± 4.01	28.41 ± 3.86	32.74 ± 3.16	<0.001	1.23

**Table 2:** Partial correlations (controlling for sex) for firefighter trainees from one department for the Illinois agility test (IAT) with metronome push-ups, pull-ups, 4.54-kg backwards overhead medicine ball toss (BOMBT), leg tuck, estimated maximal aerobic capacity from the 20-m multistage fitness test (estimated VO_2max_), 10-repetition maximum (10RM) deadlift, and the farmer’s carry.

	IAT		
Variable	*r*	*p*	*r Strength*
Push-ups	–0.138*	0.019	Small
Pull-ups	–0.303*	<0.001	Small
BOMBT	–0.244*	<0.001	Small
Leg Tuck	–0.267*	<0.001	Small
Estimated VO_2max_	–0.439*	0.001	Moderate
10RM Deadlift	–0.285*	0.003	Small
Farmer’s Carry	0.310*	0.002	Moderate

* Significant (p < 0.05) relationship with the IAT.

**Table 3:** Stepwise linear regression analysis between the Illinois agility test (IAT) with metronome push-ups, pull-ups, 4.54-kg backwards overhead medicine ball toss (BOMBT), leg tuck, estimated maximal aerobic capacity from the 20-m multistage fitness test (estimated VO_2max_), 10-repetition maximum (10RM) deadlift, and the farmer’s carry in male and female firefighter trainees from one department (n = 292).

Variables	*R*	*R* ^2^	Adjusted *R*^2^	*F*	*p*	Beta Coefficient
Sex	0.351	0.123	0.120	40.705	<0.001	0.351
Sex Estimated VO_2max_	0.540	0.292	0.287	59.558	<0.001	0.243 –0.425
Sex Estimated VO_2max_ 10RM Deadlift	0.596	0.355	0.348	52.758	<0.001	0.125 –0.414 –0.279
Sex Estimated VO_2max_ 10RM Deadlift BOMBT	0.622	0.387	0.379	45.329	<0.001	0.005 –0.415 –0.238 –0.229
Sex Estimated VO_2max_ 10RM Deadlift BOMBT Farmer’s Carry	0.631	0.398	0.388	37.881	<0.001	0.003 –0.395 –0.192 –0.205 0.126

## Discussion

This study investigated the relationships between different measures of fitness and the IAT, which was used as a measure of COD speed, in firefighter trainees. Firstly, the data demonstrated that, in general, male trainees outperformed female trainees in all fitness tests. Previous first responder research has shown males tend to perform better than females in general fitness tests (Cesario et al., 2018; Lockie et al., 2022), which can be, in part, linked to general sex-specific differences in body size (Fryar et al., 2016) and muscle mass (Janssen et al., 2000). These results provide support to recommendations that female firefighter trainees may require individualized strength and conditioning programs to develop their anaerobic and aerobic capacity (Lockie et al., 2022), and for fire departments who implement specific pre-academy training programs specifically targeting women (Los Angeles County Fire Department, n.d.). Relative to the primary focus of this study, the results indicated that all fitness tests correlated with the IAT performed by firefighter trainees in the current sample. As will be discussed, these results may influence the fitness training program design for trainees during the academy period, especially as it pertains to COD speed. This could then impact the firefighter’s ability to efficiently and safely maneuver around the fire ground. The results from this study could also be applied to athletes from sports that need to develop their COD.

As stated, COD speed incorporates linear speed, movement technique, strength and power, as well as deceleration and acceleration capacity (Fernandez-Fernandez et al., 2022; Lockie et al., 2016, 2019; Sheppard and Young, 2006). Firefighting-specific ability tests such as the BPAT, which simulates firefighting job tasks, incorporates COD movements (Santa Ana College Fire Technology Department, 2021), providing an indication of the importance of this quality for firefighters. The correlation results showed that all fitness tests correlated with the IAT, although the strength of these relationships was small-to-moderate. These results may be representative of previous research in law enforcement recruits, whereby individuals who are generally fitter tended to perform well in a range of different fitness tests (Cesario et al., 2018; Lockie et al., 2018). The IAT correlation data may also highlight why COD speed tests may be used to provide a metric for multiple fitness qualities in occupational testing (Cesario et al., 2018; Lockie et al., 2018). Nonetheless, the results suggest that firefighter trainees who are faster in a COD speed test such as the IAT may also have better muscular strength, power, as well as anaerobic and aerobic capacity. Improving the general conditioning of firefighter trainees could theoretically improve their COD speed. The stepwise regression analyses investigated this concept further to potentially provide more specific recommendations.

In addition to trainee sex, the IAT was predicted by estimated VO_2max_ (aerobic capacity), 10RM deadlift (lower-body muscular strength), BOMBT (total-body power), and the farmer’s carry (anaerobic capacity). Previous research has shown that lower-body power and strength are important for faster COD speed as measured by the IAT in college-aged individuals (Lockie et al., 2019); the current results support these assertions in firefighter trainees. Improving strength and power could contribute to improved COD ability in firefighter trainees, which could enhance their ability to move more efficiently about the fire ground. The average IAT time attained by the trainees from this study (18.40 ± 1.19 s) underscores why aerobic and anaerobic capacity could contribute to a faster test time. An IAT test of this duration could make an individual’s metabolic capacity the limiting factor in test performance (Vescovi and McGuigan, 2008). This would place greater stress on aerobic and anaerobic capacity. It should be noted that the explained variance was only 39%, which implies other factors not measured in the test battery could have contributed to a faster IAT (e.g., movement technique, unilateral lower-body power, etc.). Nonetheless, improving absolute strength, power, and metabolic capacity could improve a firefighter trainee’s performance in longer COD speed tasks. This would also have application to athletes who require longer COD speed in their sports (Lockie, 2019; Lockie et al., 2019).

There are study limitations that warrant notice. This study involved an analysis of archival data, so the researchers did not have input into the selection of fitness tests. Accordingly, only one measure of COD speed was measured in this study (i.e., the IAT), and there are numerous tests that could be used to measure this fitness quality (Lockie, 2019). Additionally, despite its use in previous public safety studies (Adams et al., 2014; Lockie et al., 2022), more research is required to analyze the importance of the IAT relative to factors such as job performance and academy graduation in trainees. Demographic information about the trainees (i.e., age, height, and body mass) were not provided to the researchers which may limit comparisons across other studies on firefighter populations. The tests were not completed in the order typically recommended by organizations such as National Strength and Conditioning Association (McGuigan, 2015), which could have affected the collected data.

Within the context of these limitations, this study showed that better performance on numerous fitness tests (i.e., push-ups, pull-ups, leg tucks, estimated VO_2max_, BOMBT, 10RM deadlift, and farmer’s carry) correlated with faster performance in the IAT. These results may be indicative of trainees who are generally fitter performing well in a range of different fitness tests. These results are also notable given the body of literature regarding the importance of multiple physical fitness components and effective firefighter occupational task performance. To this end, the IAT could be used to document the COD speed and ability of firefighter personnel. Furthermore, and in support of the correlation analysis, the stepwise regression analysis indicated that in addition to trainee sex, muscular strength (10RM deadlift), total-body power (BOMBT), and metabolic capacity (estimated VO_2max_, farmer’s carry) could predict IAT performance. Improving these qualities via specific strength and conditioning programs could enhance COD speed and improve a firefighter’s ability to move efficiently around the fire ground.
